# Identification of Risk of QT Prolongation by Pharmacists When Conducting Medication Reviews in Residential Aged Care Settings: A Missed Opportunity?

**DOI:** 10.3390/jcm8111866

**Published:** 2019-11-04

**Authors:** Louise Christensen, J. Rick Turner, Gregory M. Peterson, Mark Naunton, Jackson Thomas, Kwang Choon Yee, Sam Kosari

**Affiliations:** 1 Discipline of Pharmacy, Faculty of Health, University of Canberra, Bruce, ACT 2617, Australia; louiseclairechristensen@hotmail.com (L.C.); g.peterson@utas.edu.au (G.M.P.); Mark.Naunton@canberra.edu.au (M.N.); Jackson.Thomas@canberra.edu.au (J.T.); Kwang.Yee@canberra.edu.au (K.C.Y.); 2 Department of Pharmacy Practice, Campbell University College of Pharmacy & Health Sciences, 239 J.P. Riddle Building, PO Box 1090, Buies Creek, NC 27506, USA; j.rick.turner123@outlook.com; 3 Faculty of Health, University of Tasmania, Hobart 7005, Tasmania, Australia

**Keywords:** QT interval prolongation, torsade de pointes, residential aged care facilities, nursing homes, pharmacist, medication review, elderly

## Abstract

QT interval prolongation is associated with torsade de pointes and sudden cardiac death. QT prolongation can be caused by many drugs that are commonly prescribed in elderly residential aged care populations. The aim of this study was to investigate the prevalence of use of QT-prolonging drugs and to identify interventions made by pharmacists to reduce the risk of QT prolongation when conducting medication reviews in aged care. A retrospective analysis of 400 medication reviews undertaken by Australian pharmacists in aged care settings was conducted. The assessment included the risk of QT prolongation due to prescribed medications and other risk factors and the recommendations made by pharmacists to reduce the risk of QT prolongation. There was a high prevalence of the use of QT-prolonging medication, with 23% of residents (92 out of 400) taking at least one medication with a known risk of QT prolongation. Amongst the 945 prescribed drugs with any risk of QT prolongation, antipsychotics were the most common (*n* = 246, 26%), followed by antidepressants (19%) and proton pump inhibitors (13%). There appeared to be low awareness amongst the pharmacists regarding the risk of QT prolongation with drugs. Out of 400 reviews, 66 residents were categorised as high risk and were taking at least one medication associated with QT prolongation; yet pharmacists intervened in only six instances (9%), mostly when two QT-prolonging medications were prescribed. There is a need to increase awareness amongst pharmacists conducting medication reviews regarding the risk factors associated with QT prolongation, and further education is generally needed in this area.

## 1. Introduction

The QT interval is defined as the time between the onset of the QRS complex and the offset of the T wave, as seen on the surface electrocardiogram (ECG), and represents ventricular depolarisation and repolarisation, i.e., the time between contraction and relaxation of the heart muscle [1]. The QT interval in healthy adult males and females is typically <430 msec and <450 msec, respectively [2]. A prolonged QT interval (>450 msec for males and >470 msec for females) is associated with an increased risk of ventricular arrhythmias, including the potentially lethal torsade de pointes (TdP) [2].

TdP is a rare polymorphic ventricular arrhythmia that typically occurs in self-limiting bursts leading to symptoms of dizziness, palpitations, syncope, and seizures, but can occasionally progress to ventricular fibrillation and sudden cardiac death [3]. The electrocardiographic waveform of TdP is characterised by rapid irregular QRS complexes that appear to twist around the isoelectric baseline. It can result from having inherited long QT syndrome (LQTS) or acquired conditions such as drug-induced QT prolongation [4]. The latter observation led Link and colleagues to note that “one of the most feared complications in medicine is sudden death caused by drug-induced proarrhythmia” [5]. Many medications commonly prescribed in residential aged care populations may prolong the QT interval directly or indirectly. These include antipsychotics, antidepressants, and medications for cardiac conditions [6].

The QT interval can also be impacted by many clinical conditions, such as thyroid disturbances, diseases of the cardiovascular system (e.g., hypertension, atrial fibrillation, bradycardia and conduction disease, structural heart disease, heart failure, and ischaemic cardiomyopathy), hypokalaemia, hypocalcaemia, hypochloraemia, and hypomagnesaemia, neurological and metabolic disturbances, and renal and liver impairment [7,8,9,10]. Apart from genetic factors, patient demographics such as older age (≥65 years) and female gender are also risk factors for QT prolongation [10].

In residential aged care facilities, patients with multiple risk factors for QT prolongation, including the use of QT-prolonging drugs, are common [6]. Therefore, pharmacists working in the aged care sector can play a pivotal role in the recognition of high-risk patients and management of drugs associated with QT prolongation.

In Australia, residential medication management reviews (RMMRs) are the primary service for promoting the quality use of medicines in aged care [11,12,13]. Collaborative RMMRs are defined as “resident-focused, collaborative, comprehensive medication reviews involving the systematic evaluation of the resident’s complete medication regimen and management of that medicine in the context of other relevant clinical information and the resident’s health status” [13]. RMMRs are provided by accredited pharmacists and may include participation from general medical practitioners, aged care nursing staff, and residents and their family members; they have been remunerated since 1997. In general, only one RMMR can be performed annually for each resident. To be eligible to receive government reimbursement for undertaking medication reviews, pharmacists must achieve and maintain a qualification additional to their license to practice. Pharmacists must be accredited with either of two bodies responsible for training and assessing accredited pharmacists (Australian Association of Consultant Pharmacy or the Society of Hospital Pharmacists of Australia). To obtain accreditation with either body, a pharmacist must be registered to practice as a pharmacist in Australia and successfully complete a variety of competency-based assessments. When performing medication reviews, the accredited pharmacists are not required to conduct standardised checks (e.g., for medications using explicit criteria for inappropriate prescribing or electronic tools to detect potential drug–drug interactions). The procedure varies amongst individual pharmacists, and the ability to detect potential drug-related problems is inevitably dependent on the individual’s knowledge and experience.

An earlier publication from our research group [14] addressed the identification of community-dwelling patients at risk of QT interval prolongation during medication reviews, and concluded that there is a need to improve knowledge and awareness of this topic amongst pharmacists performing such medication reviews. This complementary research explored the role of pharmacists in reducing the risk of QT prolongation in residential aged care patients by conducting medication reviews. The aim was to investigate the prevalence of the use of QT-prolonging drugs in residential aged care facility residents and to identify the interventions made by pharmacists to reduce the risk of QT prolongation when conducting RMMRs.

## 2. Materials and Methods

A sample of 1000 de-identified RMMR reports was obtained from a clinical pharmacy service provider. The reports were performed by nine accredited pharmacists during the period of 2013–2015. We randomly selected (using computer-generated random numbers) and analysed 400 of these reports retrospectively. Data including patient demographics, clinical and medication information, and the pharmacists’ recommendations were extracted and entered into a spreadsheet. In particular, the researchers could determine clinical risk factors for QT prolongation and the use of QT-prolonging medications.

The website Crediblemeds.org [15] was used by the researchers for a list of drugs that are associated with an increased risk of QT prolongation. A scientific review board, based on ongoing and systematic analysis of evidence, categorised drugs into three categories based on their ability to cause QT prolongation or TdP [14]. These categories are (1) known risk of TdP—drugs that prolong the QT interval and are clearly associated with a known risk of TdP, even when taken as recommended; (2) possible risk of TdP—drugs that can cause QT prolongation but currently lack evidence for a risk of TdP when taken as recommended; and (3) conditional risk of TdP—drugs that are associated with TdP but only under certain conditions or by creating conditions that facilitate or induce TdP [14]. The website also has a fourth category, drugs that should be avoided in individuals with congenital long QT syndrome; however, this category was not included in the study.

The RISQ-PATH score is a validated tool used to predict the risk of QT prolongation [16]. It has two elements and is calculated by the sum of (1) identified risk factors based on evidence, with points allocated on the strength of that evidence; and (2) QT-prolonging drugs, with each being allocated points based on the associated risk. A RISQ-PATH score of <10 was considered a low risk of QT prolongation whilst a RISQ-PATH score of ≥10 was considered a high risk [16]. Table 1 shows the associated risk factors and allocated points in accordance with the RISQ-PATH score. It should be noted that some risk factors were not included in the overall RISQ-PATH score due to lack of available or consistently reported data (i.e., smoking status, body mass index, and prolonged QT interval at baseline ECG).

## 3. Results

The characteristics of the 400 patients are presented in Table 2 and the most commonly prescribed QT-prolonging medications are shown in Table 3. Overall, 92% (369/400) of residents were taking at least one medication associated with a risk of QT prolongation (i.e., known, possible, or potential risk). There were 92 (23%) residents taking at least one drug with known risk of QT prolongation. The most commonly prescribed drugs with a known risk of QT prolongation in this sample of aged care residents were antidepressants (escitalopram and citalopram) (*n* = 42 residents, 10%), donepezil (*n* = 19 residents, 5%), and haloperidol (*n* = 18 residents, 5%). The most commonly prescribed combinations of drugs that are associated with a risk of QT prolongation, with at least one drug from the known risk category, were escitalopram and furosemide (*n* = 11 residents, 3%), escitalopram and risperidone (*n* = 8 residents, 2%), and donepezil and furosemide (*n* = 5 residents, 1%). Amongst the 945 prescribed drugs with any risk of QT prolongation, antipsychotics were the most common (*n* = 246, 26%), followed by antidepressants (19%), and proton pump inhibitors (PPIs; 13%).

**Figure 1:**
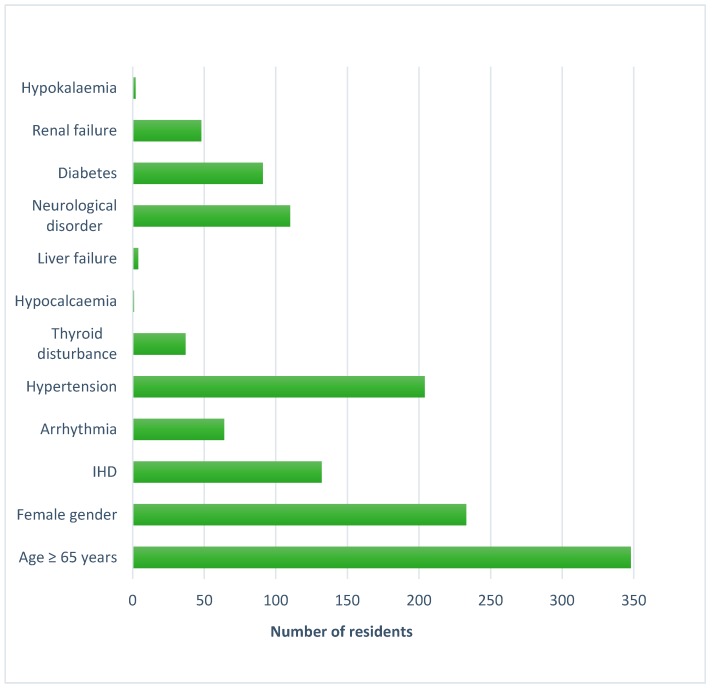
Prevalence of risk factors for drug-induced QT prolongation. IHD, Ischaemic heart disease.

Age ≥65 years was the most prevalent non-pharmacological risk factor for QT prolongation. The prevalence of other risk factors is shown in Figure 1. Forty-eight percent (*n* = 193) of residents had a high risk of QT prolongation (RISQ-PATH score of ≥10), of whom 77% (*n* = 149) were female. 

Analysing the recommendations of pharmacists in RMMR reports showed that QT prolongation was only directly identified as an issue of concern a total of 17 times across the 400 residents, 8 of which were in high-risk patients and 9 in low-risk patients. The majority of the high-risk patients (66% (*n* = 127 out of 193)) were not taking any drugs from the known-risk category; rather, they were taking a combination of possible- and conditional-risk drugs.

More pertinently, 16% (*n* = 66 out of 400) of the study sample were taking at least one drug with a known risk of QT prolongation and had a RISQ-PATH score of ≥10. Hence, there were potentially 66 instances where a pharmacist intervention could have been made. Pharmacists identified the risk of QT prolongation in only 9% (*n* = 6) of these cases. In these instances, pharmacists made specific recommendations of a dose reduction in 33.3% (*n* = 2) of cases, unspecific recommendations of assess/monitor/review in 33.3% (*n* = 2) of cases, and no recommendation at all in 33.3% (*n* = 2) of cases.

Thirty-four percent (*n* = 23 out of 66) of these high-risk residents were taking one drug from the known-risk category and had a RISQ-PATH score of ≥15; in all those residents, pharmacists did not identify the risk of QT prolongation. However, five residents with a RISQ-PATH score of ≥10 were taking two drugs from the known-risk category (amiodarone and escitalopram, citalopram and haloperidol, domperidone and citalopram), and thus were considered at highest risk of QT prolongation in the study sample. In those residents, pharmacists identified the risk of drug-induced QT prolongation in 40% (*n* = 2 out of 5) of the medication reviews.

## 4. Discussion

Overall, 16% of the sample were at a high risk of QT prolongation and were taking at least one medication with a known risk of QT prolongation. Citalopram and escitalopram were the most commonly prescribed drugs with a known risk of QT prolongation. Antipsychotics, antidepressants, and PPIs were the most commonly prescribed drug groups with any risk of QT prolongation in this sample.

Polypharmacy in elderly populations is well established and increases the risk of adverse drug reactions (ADRs). Pharmacist involvement and intervention is key to reducing drug-related misadventure and to optimising patient care [17]. Inappropriate use and overuse of drugs such as antipsychotics, antidepressants, and PPIs in older adults—especially aged care residents—have been reported in many studies [18,19,20]. However, research specifically looking at the risk of QT prolongation in residents in residential aged care facilities has been lacking.

The large number of QT-prolonging drugs that are prescribed to residents in aged care facilities, combined with the prevalence of risk factors, such as age ≥65 years, diseases of the cardiovascular system, neurological disturbances, age-related renal decline, and electrolyte imbalances, suggests that these residents are at a much higher risk of QT prolongation. Despite this, relatively few interventions were recommended by pharmacists, and related recommendations were mainly targeted at drugs with known risk of QT prolongation—especially in cases when two drugs with known risk of QT prolongation were concomitantly used, rather than patients’ other risk factors.

Most prescribers commonly encounter warnings about drugs that can cause QT prolongation. However, it may be challenging for clinicians to weigh the potential risk of QT prolongation against the benefit of a drug [21]. It is particularly difficult for a pharmacist to recommend the removal of a therapy that appears to be effective and may be considered important for the ongoing management of the patient (e.g., donepezil in early stage dementia). An observational study in Belgian community pharmacies reported that when pharmacists contacted prescribers regarding QT-prolonging drug interactions and proposed interventions, in more than half of the cases the proposed interventions were considered unnecessary by the prescribers [22].

Our study adds to a growing body of research showing that there is a need for greater awareness amongst pharmacists and other health professionals regarding the risk factors—both pharmacological and non-pharmacological—associated with QT prolongation, and hence identification of patients at greatest risk of TdP [23,24,25]. Several educational review papers are now available and suitable for this purpose [4,26,27,28].

With regard to educational activities providing a greater awareness amongst multiple healthcare professions about the risk factors associated with QT prolongation, Turner [29] published an editorial in 2018 entitled: “Proposed proarrhythmic cardiac safety education in medical, pharmacy, and nursing schools: An interprofessional model.” While teaching students and practitioners of each of these disciplines about drug-induced QT prolongation and proarrhythmic cardiac safety is a very worthwhile goal, an interprofessional model takes educational endeavours to an even higher level. The safe practice of pharmaceutical medicine involves participation of all of these disciplines. Physicians who are considering prescribing a QT-prolonging medicine need to make a careful analysis of the patient’s other risk factors for drug-induced prolongation. Second, given their expert knowledge of clinical pharmacology, pharmacists are well-placed to work closely with physicians as influential arbiters of sound prescribing decisions, and to alert physicians to injudicious prescribing decisions (although it will be important to educate prescribing physicians in the value of such work by pharmacists). Third, nurses in inpatient and residential care settings are well-placed to conduct a final check before a prescribed and dispensed medicine is administered to a patient [29]. Additionally, Klotzbaugh and colleagues [28] expanded this line of thinking to include physician assistants. Collectively, these papers can be regarded as an initial set of resources for educating multiple healthcare professions about proarrhythmic safety in an interprofessional manner.

The practical use of such resources could occur in several settings. For example, in academic institutions that have more than one healthcare professional school, joint workshops and/or symposia provide the opportunity for initial didactic teaching followed by interprofessional discussions involving students and faculty from each professional school on campus. The papers by Thind and colleagues [27] and Klotzbaugh [28] may be particularly useful for generating such interprofessional discussions, as they include clinical vignettes specifically designed for this purpose. Each of the vignettes addresses how an individual patient was prescribed certain drug(s), and highlights the effects of these prescriptions. Expert commentary is provided to illustrate “lessons learned” that are valuable to all healthcare professions addressed here.

A second practical implementation of published resources is the education of professionals already in clinical practice; continuing education initiatives in proarrhythmic cardiac safety can be very valuable. One example, a paper by Turner and colleagues [4], is being used for continuing education in medicine and pharmacy, and it is hoped that such activities will also be created for the nursing and physician assistant professions.

There are limitations to this study. The data available in the RMMRs for some of the risk factors were limited and therefore there were potentially more patients who could have been classified as being at high risk if all the data were available. We also did not have clinical outcome data for the residents, such as QT intervals or the incidence of QT prolongation. The existing system and the risk calculation tool (RISQ-PATH) adopted for this study were largely based on a limited number of relatively small observational studies that categorized the risk based on the potential of a factor or a drug to cause QT prolongation. It is possible that a previous annual medication review addressed QT prolongation. A relatively small number of accredited pharmacists performed the reviews. Finally, awareness amongst pharmacists conducting medication reviews regarding the risk factors associated with QT prolongation may have improved since 2015.

## 5. Conclusions

It appears that accredited pharmacists in Australia are not routinely evaluating QT prolongation risk when performing medication reviews in aged care settings, despite residents in aged care facilities being at an appreciable risk of drug-induced QT prolongation and potentially TdP. There is a need for greater awareness amongst pharmacists conducting medication reviews regarding drug-induced QT prolongation and other risk factors associated with QT prolongation.

## Figures and Tables

**Table 1:** Risk factors for QT prolongation according to RISQ-PATH score [15].

Risk Factor	Points Allocated
Age ≥65 years	3
Female gender	3
Smoking	3
Body mass index ≥30 kg/m^2^	1
Ischaemic cardiomyopathy	3
Hypertension	3
Arrhythmia	3
Prolonged QT interval on baseline ECG	6
Thyroid disturbance	3
Liver failure	1
Neurological disorder (stroke, trauma, infection, tumour)	0.5
Diabetes	0.5
Hypokalaemia (potassium ≤3.5 mmol/L)	6
Hypocalcaemia (calcium <2.15 mmol/L)	3
CRP >5mg/L (inflammation)	1
Renal impairment (eGFR ≤30 mL/min/1.73 m^2^)	0.5
For each known risk category 1 drug	3
For each possible risk category 2 drug	0.5
For each conditional risk category 3 drug	0.25
Total RISQ-PATH score	Max 40.5 + sum QT drugs

CRP, C-reactive protein; ECG, electrocardiogram.

**Table 2:** Patient characteristics.

Characteristics	Mean ± SD (Range) or *n* (%)
Number of residents	400
Age, years	79 ± 13 (37–101)
Female	233 (58%)
Number of medications per resident Number of medications with known risk of QT prolongation per resident Number of medications with possible risk of QT prolongation per resident Number of medications with conditional risk of QT prolongation per resident	12 ± 4 (0–26) 0.2 ± 0.5 (0–2) 1 ± 0.8 (0–4) 1 ± 1.1 (0–5)
Number of medical conditions per resident	10 ± 4 (1–26)
Mean RISQ-PATH score per resident	9.5 ± 4 (0–25.75)

SD, standard deviation.

**Table 3:** Use of QT-prolonging medications.

“Known Risk”	Number of Residents	% of Total Number of Medications	“Possible Risk”	Number of Residents	% of Total Number of Medications	“Conditional Risk”	Number of Residents	% of Total Number of Medications
escitalopram	21	2.2	risperidone	73	7.7	furosemide	108	11.4
citalopram	21	2.2	mirtazapine	65	6.9	metoclopramide	92	9.7
donepezil	19	2.0	buprenorphine	46	4.9	quetiapine	71	7.5
haloperidol	18	1.9	memantine	25	2.6	esomeprazole	61	6.5
amiodarone	6	0.6	tramadol	23	2.4	olanzapine	56	5.9
domperidone	4	0.4	venlafaxine	16	1.7	pantoprazole	34	3.6
ondansetron	2	0.2	aripiprazole	12	1.3	sertraline	30	3.2
sotalol	2	0.2	lithium	11	1.2	omeprazole	26	2.8
fingolimod	1	0.1	clozapine	8	0.8	loperamide	22	2.3
flecainide	1	0.1	promethazine	4	0.4	paroxetine	11	1.2
ketoconazole	1	0.1	imipramine	1	0.1	galantamine	11	1.2
TOTAL	96	10	paliperidone	1	0.1	hydrochlorothiazide	10	1.1
			tamoxifen	1	0.1	amitriptyline	9	1.0
			tetrabenazine	1	0.1	indapamide	7	0.7
			tolterodine	1	0.1	amisulpride	4	0.4
			TOTAL	288	30	solifenacin	3	0.3
						amantidine	2	0.2
						fluoxetine	1	0.1
						hydroxychloroquine	1	0.1
						metronidazole	1	0.1
						lansoprazole	1	0.1
						TOTAL	561	59
